# Trends in Venous Thromboembolism Anticoagulation in Patients Hospitalized With COVID-19

**DOI:** 10.1001/jamanetworkopen.2021.11788

**Published:** 2021-06-11

**Authors:** Valerie M. Vaughn, Monica Yost, Chelsea Abshire, Scott A. Flanders, David Paje, Paul Grant, Scott Kaatz, Tae Kim, Geoffrey D. Barnes

**Affiliations:** 1 Division of General Internal Medicine, Department of Internal Medicine, University of Utah, Salt Lake City; 2 Division of Health System Innovation & Research, Department of Population Health Science, University of Utah, Salt Lake City; 3 Division of Hospital Medicine, Department of Internal Medicine, University of Michigan, Ann Arbor; 4 Michigan Value Collaborative, Department of Surgery, University of Michigan, Ann Arbor; 5 Division of Hospital Medicine, Henry Ford Hospital, Detroit, Michigan; 6 Michigan Arthroplasty Registry Collaborative Quality Initiative, Department of Orthopedic Surgery, University of Michigan, Ann Arbor; 7 Division of Cardiovascular Medicine, Department of Internal Medicine, University of Michigan, Ann Arbor

## Abstract

**Importance:**

Venous thromboembolism (VTE) is a common complication of COVID-19. It is not well understood how hospitals have managed VTE prevention and the effect of prevention strategies on mortality.

**Objective:**

To characterize frequency, variation across hospitals, and change over time in VTE prophylaxis and treatment-dose anticoagulation in patients hospitalized for COVID-19, as well as the association of anticoagulation strategies with in-hospital and 60-day mortality.

**Design, Setting, and Participants:**

This cohort study of adults hospitalized with COVID-19 used a pseudorandom sample from 30 US hospitals in the state of Michigan participating in a collaborative quality initiative. Data analyzed were from patients hospitalized between March 7, 2020, and June 17, 2020. Data were analyzed through March 2021.

**Exposures:**

Nonadherence to VTE prophylaxis (defined as missing ≥2 days of VTE prophylaxis) and receipt of treatment-dose or prophylactic-dose anticoagulants vs no anticoagulation during hospitalization.

**Main Outcomes and Measures:**

The effect of nonadherence and anticoagulation strategies on in-hospital and 60-day mortality was assessed using multinomial logit models with inverse probability of treatment weighting.

**Results:**

Of a total 1351 patients with COVID-19 included (median [IQR] age, 64 [52-75] years; 47.7% women, 48.9% Black patients), only 18 (1.3%) had a confirmed VTE, and 219 (16.2%) received treatment-dose anticoagulation. Use of treatment-dose anticoagulation without imaging ranged from 0% to 29% across hospitals and increased over time (adjusted odds ratio [aOR], 1.46; 95% CI, 1.31-1.61 per week). Of 1127 patients who ever received anticoagulation, 392 (34.8%) missed 2 or more days of prophylaxis. Missed prophylaxis varied from 11% to 61% across hospitals and decreased markedly over time (aOR, 0.89; 95% CI, 0.82-0.97 per week). VTE nonadherence was associated with higher 60-day (adjusted hazard ratio [aHR], 1.31; 95% CI, 1.03-1.67) but not in-hospital mortality (aHR, 0.97; 95% CI, 0.91-1.03). Receiving any dose of anticoagulation (vs no anticoagulation) was associated with lower in-hospital mortality (only prophylactic dose: aHR, 0.36; 95% CI, 0.26-0.52; any treatment dose: aHR, 0.38; 95% CI, 0.25-0.58). However, only the prophylactic dose of anticoagulation remained associated with lower mortality at 60 days (prophylactic dose: aHR, 0.71; 95% CI, 0.51-0.90; treatment dose: aHR, 0.92; 95% CI, 0.63-1.35).

**Conclusions and Relevance:**

This large, multicenter cohort of patients hospitalized with COVID-19, found evidence of rapid dissemination and implementation of anticoagulation strategies, including use of treatment-dose anticoagulation. As only prophylactic-dose anticoagulation was associated with lower 60-day mortality, prophylactic dosing strategies may be optimal for patients hospitalized with COVID-19.

## Key Points

**Question:**

What is the frequency with which patients hospitalized with COVID-19 are treated with venous thromboembolism (VTE) prophylactic- and treatment-dose anticoagulation, and what is the association of anticoagulation with in-hospital and 60-day mortality?

**Findings:**

In this cohort study of 1351 patients hospitalized with COVID-19 in which 1127 patients received anticoagulation, 34.8% missed 2 or more days of VTE prophylaxis. Use of only prophylactic-dose or treatment-dose anticoagulation was associated with lower in-hospital mortality vs no anticoagulation; however, only prophylactic-dose anticoagulation remained associated with lower mortality at 60 days.

**Meaning:**

These findings suggest that prophylactic-dose VTE anticoagulation may be optimal therapy for patients hospitalized with COVID-19.

## Teaser

This cohort study of patients hospitalized with COVID-19 examines the association of anticoagulation treatment with mortality rates, and assesses differences between prophylactic- and treatment-doses in in-hospital and 60-day mortality.

## Introduction

Venous thromboembolism (VTE) has been a leading complication of COVID-19.^1^ Early publications of high VTE rates likely influenced clinical practice related to VTE prophylactic- and treatment-dose anticoagulation. First, there has been a concerted emphasis on VTE prophylaxis for hospitalized patients with COVID-19.^2,3,4,5^ Second, many experts have advocated for escalating doses of prophylactic anticoagulation for some patients hospitalized with COVID-19.^4,6,7^ The potential importance of these practices has been highlighted further by a 2020 retrospective study^8^ showing a potential mortality benefit with treatment- or prophylactic-dose anticoagulation. More recently, preliminary results from clinical trials have found a decrease in the combined outcome of in-hospital mortality and organ support free days with treatment-dose anticoagulation in patients outside of intensive care.^9,10^ Given these findings, we sought to better understand variation in anticoagulation practices for patients hospitalized with COVID and the relationship of anticoagulation strategies with in-hospital and 60-day mortality.

## Methods

### MI-COVID19 Consortium

MI-COVID19 is a statewide collaborative quality initiative (CQI) sponsored by Blue Cross Blue Shield of Michigan and Blue Care Network. In March 2020, hospitals joined to collect patient-level data on COVID-19 patients with a goal of improving patient care.^11^ Institutional participation in MI-COVID19 is voluntary, and was arranged through a special collaboration of hospitals participating in other Blue Cross Blue Shield–sponsored CQIs, including those with experience publishing data on VTE outcomes and anticoagulation.^12,13^ Of the 92 noncritical access, nonfederal hospitals in Michigan, 38 (41%) elected to participate in MI-COVID19. MI-COVID19 hospitals are located across Michigan and have a median bed size of 391 (interquartile range [IQR], 250-537 beds); 81% are nonprofit and 93% self-identify as teaching hospitals. There were no standardized treatment protocols provided as part of MI-COVID19. MI-COVID19 received nonregulated status prior to data collection by the University of Michigan institutional review board. This study followed the Strengthening the Reporting of Observational Studies in Epidemiology (STROBE) reporting guideline for cohort studies.

### Patient Inclusion

Our primary cohort of interest was adults hospitalized for COVID-19 from March 7, 2020, to June 17, 2020. Patients were excluded if pregnant, aged under 18 years, discharged against medical advice, assigned comfort care on hospitalization, or transferred from another hospital. For patients with multiple hospitalizations, the first was included. As we were interested in patients newly started on empirical anticoagulation, we excluded 203 patients on treatment-dose anticoagulants prior to hospitalization and 28 patients with a VTE diagnosed within 2 days of hospitalization. Given that patients with short hospitalizations are less likely to benefit from anticoagulation, we excluded 224 patients hospitalized for less than 3 days. Hospitals with fewer than 10 cases were also excluded.

Based on available data collection resources, some MI-COVID19 hospitals were able to include all COVID-19–positive patients. Other hospitals (eg, with high volumes) employed a pseudorandom sampling process to select cases. Pseudorandomization involved sorting potentially eligible discharges by timestamp of discharge and reviewing patients for inclusion in ascending order based on the minute in which they were discharged (full pseudorandom sampling procedure available in eAppendix 3 in the Supplement).^11^

### Data Collection

Experienced, professional abstractors for other Blue Cross Blue Shield CQIs were retrained to collect data on COVID-19 patients for MI-COVID19.^14^ Using standardized data templates and detailed data dictionaries, abstractors collected demographic data; comorbidities; daily treatment, laboratory, and stability data (eg, respiratory support); and outcomes (eg, mortality). Outcomes were also collected prospectively up to 60 days after hospitalization via medical record review and a postdischarge patient phone call (see Data Collection Outcomes). As disparities because of patient demographic characteristics may exist, we have reported data on sex, race, and ethnicity obtained from medical records and categorized as noted in eAppendix 2 of the Supplement.

#### Exposures

Abstractors collected anticoagulant administration data from each day of hospitalization. To ascertain VTE prophylaxis, abstractors were asked, “Was an anticoagulant administered for VTE prophylaxis on the date indicated?” To ascertain treatment-dose anticoagulation, abstractors were asked, “Was a treatment anticoagulant administered on the date indicated?” Abstractors determined whether anticoagulation was prophylactic- vs treatment-dose based on anticoagulant selection and common treatment and prophylactic doses. For example, low molecular weight heparin given 1 or 2 times per day at 30 to 40 mg and any subcutaneous heparin injection was considered prophylactic. Intravenous heparin infusions could be prophylactic or therapeutic based on prescriber intent (see eAppendix 1 in the Supplement). Dosing of anticoagulation did not explicitly denote prescriber intent, as use of “intermediate” doses of anticoagulation were often recommended for highest-risk patients.^4,6,7^

#### Outcomes

To assess in-hospital and 60-day mortality, abstractors reviewed the medical record 60 days following discharge to determine whether the patient was deceased. If so, date of death and—if available—cause(s) of death were abstracted. If no data were available or the patient appeared to be alive 60 days following discharge, the patient was contacted by phone up to 3 times to obtain additional outcome information. If the telephone respondent noted that the patient had died since hospitalization, they were asked for date and cause of death.

To assess in-hospital and 60-day VTE events, abstractors reviewed the medical record 60 days following discharge to determine whether there was a confirmed or suspected deep vein thrombosis or pulmonary embolism noted in the medical record and, if so, collect related imaging results. Only VTEs confirmed by imaging were considered VTE events. All data were entered into the MI-COVID19 registry using a structured data collection template.

### Definitions

#### Exposures

Anticoagulant exposure was categorized into 3 groups: (1) treatment-dose anticoagulation, defined as ever having received treatment-dose anticoagulation (for prophylactic intent) while hospitalized; (2) prophylactic-dose anticoagulation, defined as only receiving prophylactic-dose anticoagulation while hospitalized; and (3) no anticoagulation, defined as receiving neither treatment nor prophylactic anticoagulation while hospitalized. In those who received any anticoagulation, we also evaluated for nonadherence to VTE prophylaxis where any day in which a patient received neither prophylactic- nor treatment-dose anticoagulation was considered nonadherent.

#### Outcomes

Our primary outcomes of interest were in-hospital or 60-day (from hospital admission) all-cause mortality. Sixty-day mortality included mortality captured by medical record review and from telephone calls 60 days following hospitalization, with events censored at 60 days after hospital admission. Our secondary outcome of interest was 60-day VTE events including pulmonary embolism or deep venous thrombosis confirmed by imaging.

### Statistical Analysis

Descriptive statistics were used to characterize the cohort. Patient characteristics were compared by anticoagulation strategy using χ^2^, multisample median (ie, Brown-Mood), or *t* tests, as appropriate. We assessed change over time in any anticoagulant use, nonadherence to VTE prophylaxis, and treatment-dose anticoagulation without imaging, using logistic regression models adjusted for hospital clustering and patient ICU status. We report adjusted odds ratios (aOR) for the effect of each additional week on anticoagulant use.

To evaluate the association of anticoagulant strategies with in-hospital and 60-day mortality, we used inverse probability of treatment models to control for variables associated with anticoagulant use. Similar to prior studies,^8^ we fit multinomial logit generalized linear models with anticoagulant exposure as the dependent variable, and age, sex, race and ethnicity, body mass index, hypertension, heart failure, chronic kidney disease, highest level of respiratory support, D-dimer (categorized as 0-2 times ULN, 2-4 times ULN, >4 times ULN, or missing) on day 1 or 2 of hospitalization, week of admission, and COVID-19 treatment (ie, IL-6 receptor inhibitor, remdesivir, and corticosteroids) as independent variables. Each patient was weighted by the inverse probability of being in their anticoagulant exposure group (for inverse probability of treatment weighting [IPTW] models see eTable 1 in the Supplement).^15^ We then used IPTW cause-specific hazard models to determine the association of anticoagulant strategy with in-hospital and 60-day mortality. Survival in days was calculated as time from hospital admission to death up to 60 days postadmission. The adjusted hazard ratios (aHR) and their respective 95% CIs are reported for all time-to-event models. A similar approach was used to evaluate the association of VTE prophylaxis adherence with mortality, where nonadherence was assessed first as a dichotomous exposure (ie, ≥2 days vs <2 days of nonadherence) and then as a continuous exposure (ie, percentage of inpatient days with nonadherence). Because low rates of 60-day VTE events, we report unadjusted event rates only.

#### Sensitivity Analyses

Because anticoagulant dosing could vary by day of hospitalization, we conducted a sensitivity analysis in which patients were categorized as prophylactic only until they had a confirmed VTE event, at which point they were categorized as treatment-dose anticoagulation. *P* values <.05 were considered significant in 2-sided tests. Analyses were completed using SAS version 9.4 (SAS Institute).

## Results

**Figure 1.**
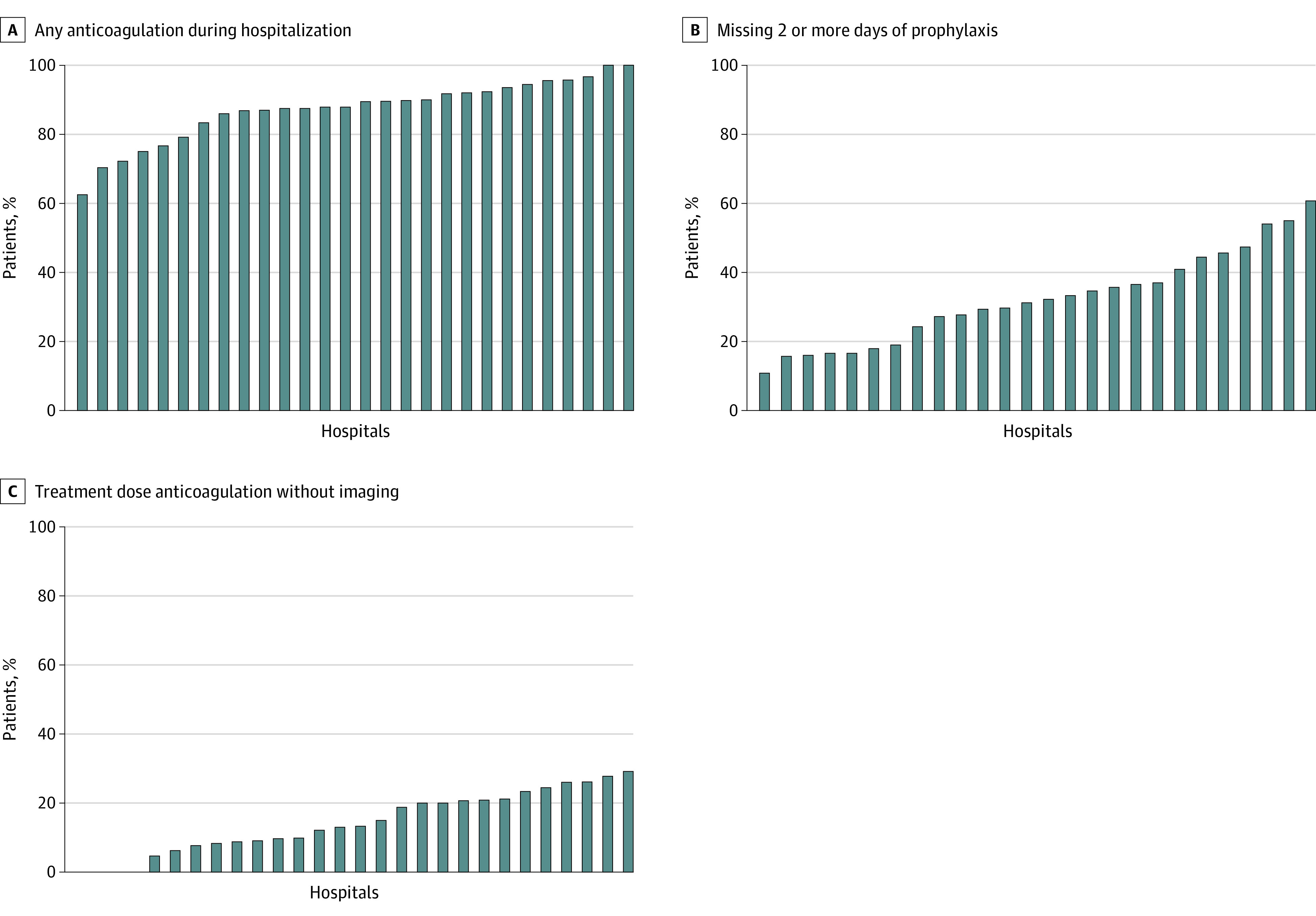
Hospital Variation in Percentage of Hospitalized Patients With COVID-19 Who Received Anticoagulation Each bar indicates 1 hospital. Subfigure B includes only patients who received prophylactic- or treatment-dose anticoagulation at some point during their hospital stay.

A total of 1351 patients with confirmed COVID-19 hospitalized in 30 hospitals were included (eFigure 1 in the Supplement for flow diagram). Included patients had a median (IQR) age of 64 (52-75) years; 645 patients (47.7%) were women, 661 (48.9%) were Black, and 540 (40.0%) were White. Median (IQR) length of stay was 6 (4-10) days and 409 patients (30.3%) received intensive care during their hospitalization. Generally, more intensive anticoagulation was given to older patients (median [IQR] age: treatment dose anticoagulants, 66 [58-76] years vs no anticoagulants, 57 [44-71] years), patients with longer lengths of stay (median [IQR] length of stay: 10 [6-15] days vs 5 [3-7] days), patients with more comorbidities (median [IQR] Charlson comorbidity score: 2 [0-3] vs 1 [0-2]), patients with more severe disease (eg, received care in an ICU: 127 patients [58.0%] vs 258 patients [26.6%]), patients who received more COVID-directed therapies (eg, corticosteroids: 111 of 209 patients [53.1%] vs 19 of 157 patients [12.1%]), and those with higher inflammatory markers (eg, median [IQR] ferritin: 848 ng/mL [376-2000] vs 597 ng/mL [296-1205]; to convert to micrograms per liter, multiply by 1.0) (Table). Only 162 patients (12.0%) received no prophylactic- or treatment-dose anticoagulation during their hospital stay (Figure 1).

**Table.** Demographic Characteristics and Laboratory Findings for Patients Testing Positive With COVID-19

Characteristics	Patients, No. (%)			*P* value
	No anticoagulants given (n = 162)	Prophylactic anticoagulation only (n = 970)	Treatment-dose anticoagulants (n = 219)	
Age, median (IQR), y	57 (44-71)	64 (53-76)	66 (58-76)	<.001
Women	89 (54.9)	469 (48.4)	87 (39.7)	.01
Men	73 (45.1)	501 (51.7)	132 (60.3)	
Race				
Black	80 (49.4)	475 (49.0)	106 (48.4)	.98
White	67 (41.4)	384 (39.6)	89 (40.7)	.89
Asian	6 (3.7)	26 (2.7)	4 (1.8)	.53
Other^a^	4 (2.5)	36 (3.7)	16 (7.3)	.03
Unknown	5 (3.1)	49 (5.1)	4 (1.8)	.08
Ethnicity				
Non-Hispanic	136 (84.0)	824 (85.0)	197 (90.0)	.13
Hispanic	10 (6.2)	53 (5.5)	9 (4.1)	.64
Unknown	16 (9.9)	93 (9.6)	13 (5.9)	.22
Skilled nursing facility prior to hospitalization	18 (11.1)	115 (11.9)	34 (15.5)	.29
Length of hospital stay, median (IQR), d	5 (3-7)	6 (4-10)	10 (6-15)	<.001
Comorbidities^b^				
History of VTE, No./No. (%)^c^	2/136 (1.5)	29/886 (3.3)	11/210 (5.2)	.16
Charlson Comorbidity Score, median (IQR)	1 (0-2)	1 (0-3)	2 (0-3)	<.001
Peripheral vascular disorders	6 (3.7)	37 (3.8)	11 (5.0)	.70
Cerebrovascular disease	14 (8.6)	98 (10.1)	31 (14.2)	.15
Cardiovascular disease	27 (16.7)	219 (22.6)	78 (35.6)	<.001
Congestive heart failure/cardiomyopathy	17 (10.5)	110 (11.3)	38 (17.4)	.04
History of myocardial infarction	9 (5.6)	41 (4.2)	17 (7.8)	.09
Moderate or severe chronic kidney disease	25 (15.4)	266 (27.4)	69 (31.1)	.002
On dialysis prior to hospitalization	5 (3.1)	29 (3.0)	7 (3.2)	.99
Moderate or severe liver disease	4 (2.5)	4 (0.4)	0 (0)	.003
Hypertension	92 (56.8)	653 (67.3)	157 (71.7)	.008
Diabetes	37 (22.8)	262 (27.0)	63 (28.8)	.42
Cancer	12 (7.4)	64 (6.6)	16 (7.3)	.89
Smoking history				
Never	105 (64.8)	588 (60.6)	100 (45.7)	<.001
Prior	39 (24.1)	272 (28.0)	86 (39.3)	.001
Current/active	10 (6.2)	51 (5.3)	13 (5.9)	.85
Unknown	8 (4.9)	59 (6.1)	20 (9.1)	.18
Severity of illness				
Received care in an ICU	24 (14.8)	258 (26.6)	127 (58.0)	<.001
Highest level of respiratory support				
No supplemental oxygen	62 (38.3)	207 (21.3)	21 (9.6)	<.001
Low-flow oxygen	86 (53.1)	563 (58.0)	81 (37.0)	<.001
Heated high-flow nasal cannula	5 (3.1)	70 (7.2)	18 (8.2)	.11
Noninvasive mechanical ventilation	0 (0)	12 (1.2)	6 (2.7)	.06
Mechanical ventilation	9 (5.6)	118 (12.2)	93 (42.5)	<.001
Required vasopressors	9 (5.6)	116 (12.0)	85 (38.8)	<.001
Required new dialysis	2 (1.2)	28 (2.9)	25 (11.4)	<.001
COVID-19 related treatments during hospitalization, No./No. (%)^d^				
Hydroxychloroquine	75/157 (47.8)	508/925 (54.9)	124/209 (59.3)	.09
Hydroxychloroquine and azithromycin	44/157 (28.0)	258/925 (27.9)	57/209 (27.3)	.98
Vitamin C (oral or intravenous)	5/157 (3.2)	126/925 (13.6)	47/209 (22.5)	<.001
Remdesivir	0/157 (0)	20/925 (2.2)	5/209 (2.4)	.17
IL-6 receptor inhibitor	1/157 (0.6)	21/925 (2.3)	22/209 (10.5)	<.001
Corticosteroids	19/157 (12.1)	247/925 (26.7)	111/209 (53.1)	<.001
Worst admission laboratory findings, median (IQR)^e^				
D-dimer, × ULN^f^	1.7 (0.9-3.3)	1.7 (1.1-3.1)	2.4 (1.2-6.9)	.04
Ferritin, ng/mL	498 (237-882)	597 (296-1205)	848 (376-2000)	.03
CRP, mg/dL	15.6 (4.8-75.7)	16.2 (6.9-72.4)	43.6 (16.2-147.2)	<.001
Creatinine, mg/dL^g^	1.0 (0.8-1.4)	1.1 (0.9-1.5)	1.2 (0.9-2.0)	<.001
Lowest platelet count, × 10^3^/μL	193 (158-258)	192 (148-247)	189 (148-252)	.71
Worst laboratory findings (entire hospitalization)				
D-dimer, × ULN^f^	1.8 (1.0-3.5)	2.0 (1.2-3.9)	6.5 (1.9-10.0)	<.001
Ferritin, ng/mL	604 (274-957)	734 (349-1435)	1233 (503-2376)	<.001
CRP, mg/dL	18.5 (7.0-75.7)	21.8 (8.7-104.0)	88.7 (21.4-203.1)	<.001
Platelet count, × 10^3^/μL				
Lowest	185 (147-250)	180 (138-237)	169 (130-232)	.12
Highest	248 (188-321)	285 (207-385)	324 (237-444)	<.001

Abbreviations: CRP, C-reactive protein; ICU, intensive care unit; IQR, interquartile range; ULN, upper limit of normal; VTE, venous thromboembolism.

SI conversion factors: To convert creatinine to micromoles per liter, multiply by 88.4; CRP to milligrams per liter, multiply by 10; ferritin to micrograms per liter, multiply by 1.0; platelet count to × 10^9^ per liter, multiply by 1.0.

^a^ Included in other were American Indian or Alaskan Native, Arab and Chaldean ancestries, Native Hawaiian or Pacific Islander, and race other than listed.

^b^ Peripheral vascular disorders include amputations from peripheral vascular disease, any arterial occlusive disease or history of a vascular surgery related to peripheral vascular disease. Moderate or severe kidney disease included history of acute or chronic kidney failure including dialysis, kidney transplantation, or creatinine levels more than 3 times the upper limit of normal. Moderate or severe liver disease included documentation of liver disease or complications of decompensated cirrhosis (eg, hepatic encephalopathy). Cancer included any nonskin solid or hematologic malignant neoplasm with or without metastasis.

^c^ Data missing for all patients with data collection prior to May 3, 2020.

^d^ Data missing for 60 patients.

^e^ Laboratory test results in the first 2 days following hospitalization.

^f^ D-dimer is reported in terms of number of times the upper limit of normal provided by the laboratory at each hospital. Given variation in reporting, values were capped at 10 times ULN.

^g^ Excludes 31 patients on dialysis.

### VTE Prophylaxis

Of 1351 total patients, 1127 (83.4%) received pharmacologic VTE prophylaxis at some point during their hospital stay and were evaluated for nonadherence to VTE prophylaxis. Subcutaneous heparin or enoxaparin injections were the most common prophylactic regimens (eTable 2 in the Supplement). Of those who ever received treatment- or prophylactic-dose anticoagulation, approximately one-third (392 of 1127 patients [34.8%]) missed 2 or more days of VTE prophylaxis.

**Figure 2.**
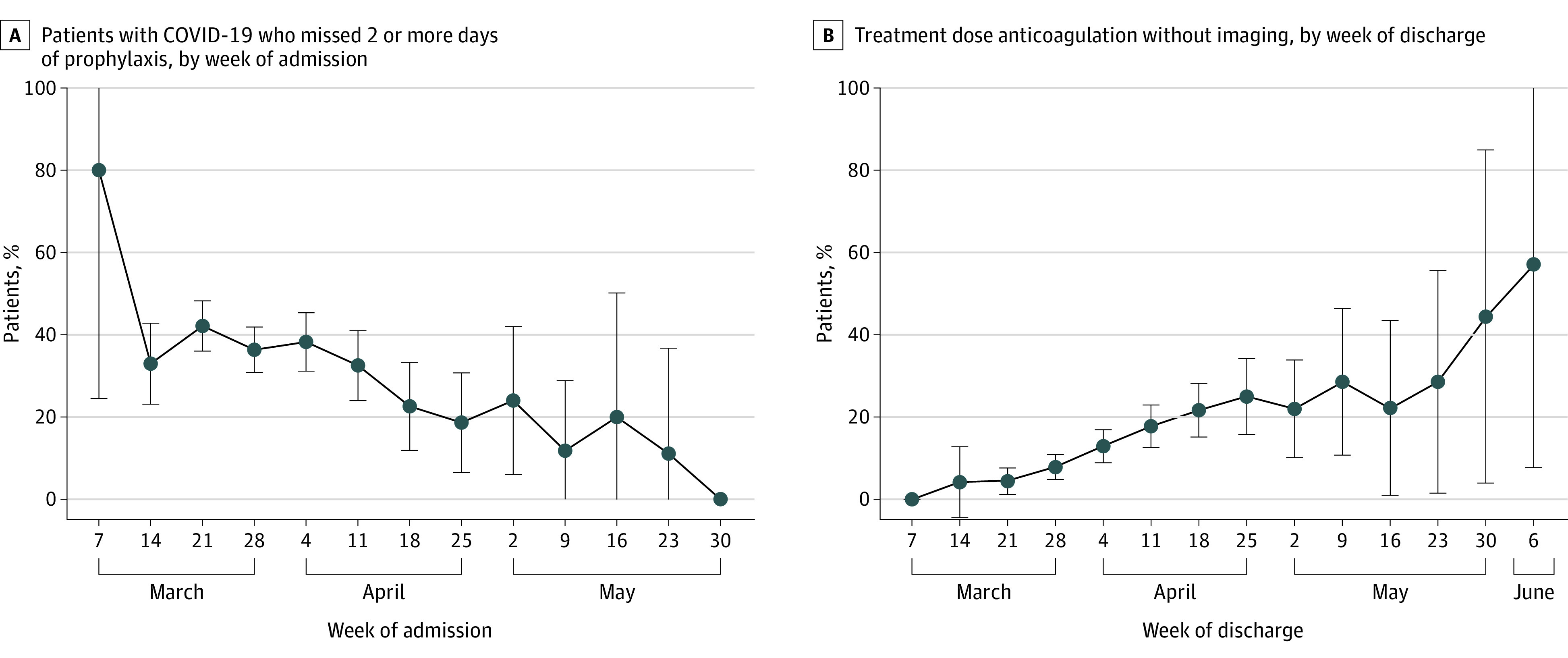
Trends in Patients Hospitalized With COVID-19 Who Received Anticoagulation by Hospitalization Dates Error bars indicate 95% CIs. After adjusting for ICU status and hospital clustering, the percentage of hospitalized patients with COVID-19 who received anticoagulation but missed 2 or more days of prophylaxis decreased over time (aOR, 0.89; 95% CI, 0.82-0.97 per week; *P* = .008). Similarly, the percentage of patients who received treatment-dose anticoagulation without imaging increased over time (aOR, 1.43; 95% CI, 1.30-1.59 per week; *P* < .001). Time starts at week 0 (March 7-14, 2020).

Nonadherence to VTE prophylaxis varied widely by hospital (from 11% to 61% of patients missing ≥2 days of VTE prophylaxis, Figure 1) and decreased markedly over time, from 80% in week 0 (March 7) to 0% by week 12 (May 30) (aOR, 0.89; 95% CI, 0.82-0.97 per week; *P* = .008) (Figure 2).

### Confirmed and Treatment-Dose VTE Treatment

Only 18 patients (1.3%) had a VTE confirmed after day 2 of hospitalization (ICU, 11 of 409 [2.7%] vs non-ICU, 7 of 942 patients [0.7%]; *P* < .004). However, 219 patients hospitalized with COVID-19 (16.2%) received treatment-dose anticoagulation for a median (IQR) 5 (3-8) days. The most common treatment-dose anticoagulants were intravenous unfractionated heparin, subcutaneous low molecular weight heparin, and oral apixaban (eTable 3 in the Supplement). ICU patients were nearly 3 times as likely to receive treatment-dose anticoagulation as general care patients (127 of 409 patients [31.1%] vs 92 of 942 patients [9.8%]; *P* < .001).

**Figure 3.**
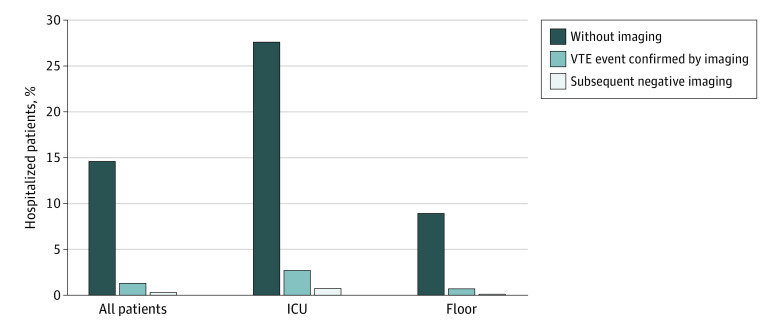
Hospitalized Patients With COVID-19 Who Received Treatment Dose Anticoagulation ICU indicates intensive care unit; VTE, venous thromboembolism.

The vast majority (197 of 219 [90.0%]) of patients who received treatment-dose anticoagulation did not have VTE diagnostic imaging (Figure 3). The percentage of patients hospitalized with COVID-19 who received treatment-dose anticoagulation without imaging varied widely by hospital, from 0% to 29% (Figure 1), and increased over time from 4% in week 1 (discharged the week of March 14th) to 57% by week 13 (discharged the week of June 6th) (aOR, 1.46; 95% CI, 1.31-1.61 per week; *P* < .001) (Figure 2).

### Mortality

Overall, 247 patients (18.3%) died while hospitalized (ICU, 168 of 409 patients [41.1%] vs general care, 79 of 942 patients [8.4%]; *P* < .001) and 313 patients (23.2%) died within 60 days of hospital admission (ICU, 184 of 409 patients [45.0%] vs general care, 129 of 942 patients [13.7%]).

In-hospital mortality (unadjusted) occurred in 12.4% (20 of 162 patients) of the no anticoagulant group, 33.8% (74 of 219 patients) of the treatment-dose anticoagulant group, and 15.8% (153 of 970 patients) of the prophylactic-dose anticoagulant group. Sixty-day mortality (unadjusted) occurred in 14.2% (23 of 162 patients) of the no anticoagulant group, 39.7% (87 of 219 patients) of the treatment-dose anticoagulant group, and 20.9% (203 of 970 patients) of the prophylactic-dose anticoagulant group (anticoagulant use on day of death in eTable 4 of the Supplement). COVID-19 was the most common documented cause of death (212 of 313 patients [67.7%]) with VTE listed as the cause of death in only 4 patients (1.3%) (eTable 5 in the Supplement).

**Figure 4.**
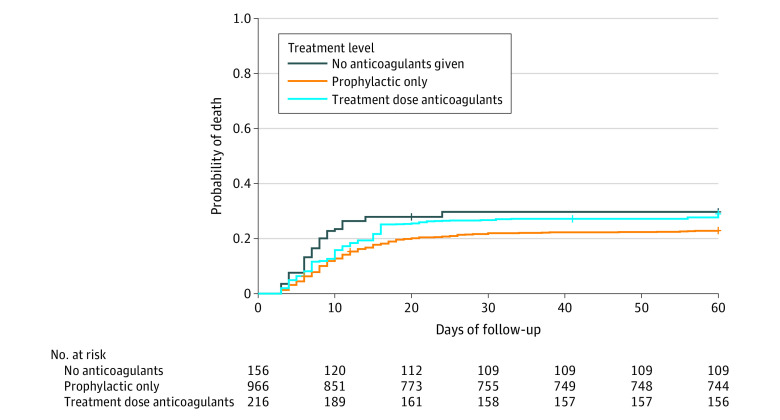
Mortality Over Time by Anticoagulant Exposure Each patient was weighted by the inverse probability of being in their anticoagulant exposure group. Compared with no anticoagulation, only prophylactic-dose anticoagulation was associated with lower mortality at 60 days (prophylactic dose: aHR, 0.706; 95% CI, 0.514-0.897; treatment dose: aHR, 0.922; 95% CI, 0.631-1.348).

After adjustments, both prophylactic- and treatment-dose anticoagulation were associated with lower in-hospital mortality compared with no anticoagulation (only prophylactic dose: aHR, 0.36; 95% CI, 0.26-0.52; ever treatment dose: aHR, 0.38; 95% CI, 0.25-0.58). However, only prophylactic-dose anticoagulation remained associated with lower 60-day mortality (prophylactic dose: aHR, 0.71; 95% CI, 0.51-0.90; treatment dose: aHR, 0.92; 95% CI, 0.63-1.35) (Figure 4). There were no statistical differences in in-hospital or 60-day mortality between patients who received treatment- vs prophylactic-dose anticoagulant therapy (inpatient mortality: aHR, 1.04; 95% CI, 0.76-1.41; 60-day mortality: aHR, 1.31; 95% CI, 0.99-1.73).

On sensitivity analysis, when patients who did not receive treatment-dose anticoagulation until after a confirmed VTE event (21 patients) were classified as prophylactic-dose only, 60-day mortality became statistically different between treatment-dose vs prophylactic-dose anticoagulation (aHR, 1.42; 95% CI, 1.07-1.88) (eTable 6 in the Supplement).

Approximately one-third (125 of 393 patients [31.8%]) of patients who had 2 or more days of VTE nonadherence experienced 60-day mortality vs 142 of 739 (19.2%) of patients without nonadherence. After adjustment, there was no significant difference in inpatient mortality (aHR, 0.86; 95% CI, 0.65-1.15); however, patients with nonadherence had a higher risk of 60-day mortality compared with patients without nonadherence (aHR, 1.31; 95% CI, 1.03-1.67) (eFigure 2 in the Supplement). Percentage of days with VTE nonadherence was not significantly associated with mortality (aHR per additional 10% nonadherence, 0.97; 95% CI, 0.91-1.03).

### VTE Events

There were confirmed 60-day VTE events in 48 patients (3.6%) (34 of 48 [70.8%] had a VTE during hospitalization). No patients in the no anticoagulation group had a 60-day VTE event, whereas 1.7% (16 of 970) and 14.6% (32 of 219) in the prophylactic- and treatment-dose groups, respectively, had a 60-day VTE (see eTable 7 in the Supplement for VTE location, eTable 8 in the Supplement for VTE timing).

## Discussion

In this large, multicenter cohort of patients hospitalized with COVID-19, use of treatment-dose anticoagulation was common, varied widely between hospitals, and increased over time. In addition to confirming recent findings suggesting both prophylactic- and treatment-dose anticoagulation strategies are associated with lower in-hospital mortality, we found that only prophylactic-dose anticoagulation was associated with lower 60-day mortality.

Critically, our study adds to existing literature on how anticoagulation strategies factor into mortality rates. First, it replicates similar findings in a retrospective, single-center New York study^8^ that found both prophylactic- and treatment-dose anticoagulation were associated with lower in-hospital mortality. We add to this by including 60-day mortality data, which demonstrated an association with lower mortality for the prophylactic-dose anticoagulation group only. One potential reason for this finding may be that treatment-dose anticoagulation prevents in-hospital death from VTE or microvascular events in some patients who then succumb from severe illness after hospital discharge. Notably, we had far more treatment-dose anticoagulation in our intensive care population (31.1% vs 9.8%) and preliminary trial data suggest treatment-dose anticoagulation improves outcomes only in non-ICU patients.^9,10^ Regardless, it appears anticoagulation is critical in hospitalized patients with COVID-19 and that prophylactic dosing may be sufficient to improve mortality.

While we found a low incidence of confirmed VTE (1.3%), the observed rate of therapeutic anticoagulation (16.2% overall, 31.1% in ICU) mirrors early reports of VTE incidence among hospitalized patients.^1^ The high and increasing use of treatment-dose anticoagulation we observed (16.2%) likely reflects increased empirical anticoagulant use for suspected VTE and increased treatment-dose anticoagulation for VTE prophylaxis. Empirical anticoagulation has been recommended by some when imaging cannot be obtained^3,4,16^; for example, when patients are too critically ill to transport or when access to imaging staff is restricted to minimize infectious exposure. At the same time, early reports of thrombosis, coagulopathy, and diffuse pulmonary microvascular thrombi as potential contributors to mortality in COVID-19 led many experts to recommend intensification of anticoagulant regimens even without evidence of thrombosis.^17^ While some clinicians and health systems use an intermediate dose of prophylaxis, others employ full therapeutic dosing. This approach is, in part, extrapolated from experience with H1N1 influenza.^18^

Given the concern about VTE risk for patients hospitalized with COVID-19, national and international efforts have strongly emphasized the need for universal use and administration of VTE pharmacologic prophylaxis.^2,3,4,5^ In our study, 12.0% of patients received no anticoagulation—potentially because they were considered low risk or had contraindications to treatment. Given what we know about coagulopathy in COVID-19 and growing evidence that anticoagulation may help hospitalized non-ICU patients the most, it is possible that even patients who seem low risk may benefit from anticoagulation. We also found evidence that adherence may be associated with 60-day mortality; prior studies have shown that missed doses of VTE prophylaxis are common and associated with higher VTE rates.^19,20,21^ Interestingly, the COVID-19 pandemic and focus on VTE prevention may represent one of the most effective implementation efforts of an evidence-based practice. In our multicenter cohort, the average number of patients with 2 or more days of missed prophylaxis medication declined from over 50% to less than 10% over just 3 months. Investigating how that implementation and dissemination effort can be replicated outside of a pandemic warrants further investigation.

Our findings have important implications. First, more data from randomized trials are needed on long-term outcomes of treatment-dose anticoagulation in patients without a confirmed VTE diagnosis. Second, VTE prophylaxis in patients with COVID-19 is standard of care. Hospitals should implement processes to ensure use of VTE prophylaxis for hospitalized patients with COVID-19. Finally, the variable and increasing use of treatment-dose anticoagulation raises concerns especially given the lack of an association with 60-day mortality. We need better methods to risk stratify and diagnose patients with VTE and a stronger evidence-base on which to decide when to employ prophylactic vs therapeutic doses of anticoagulation for patients hospitalized with COVID-19. Participation in ongoing clinical trials will help identify whether any patient groups may benefit from therapeutic doses of anticoagulation. Otherwise, given the lack of mortality difference between groups, judicious therapeutic dosing may be necessary.

### Limitations

Our findings must be taken in the context of this study’s limitations. First, as with all retrospective studies of VTE, particularly during the COVID-19 pandemic, we were limited by incomplete use of diagnostic tests for VTE. Second, we did not have bleeding outcomes, although historically this occurs in only 2% to 3% of patients.^8^ Third, we were limited in our classification of treatment- vs prophylactic-dose anticoagulation. For example, while intravenous unfractionated heparin was assumed to be therapeutic, it is possible it was given at a subtherapeutic dose and our data abstraction did not include target or actual coagulation lab tests. Fourth, anticoagulation increased and mortality decreased over time. Although we attempted to control for time, residual confounding may persist. In contrast, key strengths are inclusion of multiple hospitals, ability to assess 60-day mortality and longitudinal trends, and detailed data collection.

## Conclusions

For patients hospitalized with COVID-19, we found both prophylactic- and treatment-dose anticoagulation were associated with lower in-hospital mortality compared with no anticoagulation. Given that only prophylactic anticoagulation was associated with lower 60-day mortality, prophylactic-dose VTE prophylaxis may be the optimal therapy for patients hospitalized with COVID-19.

## Notes

Supplement. **eAppendix 1.** Abstraction Tool **eAppendix 2.** Definition of Sex, Race, Ethnicity **eAppendix 3.** COVID Case Selection/Sampling Procedure **eFigure 1.** Study Flow Diagram **eFigure 2.** 60-day Mortality Over Time by Venous Thromboembolism Prophylaxis Adherence **eTable 1.** IPTW Models **eTable 2.** Anticoagulants Given for VTE Prophylaxis, N=1127 **eTable 3.** Treatment Dose Anticoagulants, N=219 **eTable 4.** Anticoagulant Use on Day of Death N=313 **eTable 5.** Cause of 60-day Mortality, N=313 **eTable 6.** Sensitivity Analysis When 21 Patients Who Did Not Receive Treatment Dose Anticoagulation Until After a Confirmed VTE Were Classified as Prophylactic-Dose Anticoagulation Only **eTable 7.** VTE Location in Patients Who Had a 60-day VTE Event **eTable 8.** VTE Timing (Out Of All Patients)
