# Supplementary material for: Trends in Venous Thromboembolism Anticoagulation in Patients Hospitalized With COVID-19
Source: JAMA Netw Open. 2021 Jun 11;4(6):e2111788. doi: 10.1001/jamanetworkopen.2021.11788 (PMC8196344; doi:10.1001/jamanetworkopen.2021.11788)
Supplement: Supplement. — eAppendix 1. Abstraction Tool eAppendix 2. Definition of Sex, Race, Ethnicity eAppendix 3. COVID Case Selection/Sampling Procedure eFigure 1. Study Flow Diagram eFigure 2. 60-day Mortality Over Time by Venous Thromboembolism Prophylaxis Adherence eTable 1. IPTW Models eTable 2. Anticoagulants Given for VTE Prophylaxis, N=1127 eTable 3. Treatment Dose Anticoagulants, N=219 eTable 4. Anticoagulant Use on Day of Death N=313 eTable 5. Cause of 60-day Mortality, N=313 eTable 6. Sensitivity Analysis When 21 Patients Who Did Not Receive Treatment Dose Anticoagulation Until After a Confirmed VTE Were Classified as Prophylactic-Dose Anticoagulation Only eTable 7. VTE Location in Patients Who Had a 60-day VTE Event eTable 8. VTE Timing (Out Of All Patients) [file jamanetwopen-e2111788-s001.pdf]

Supplemental Online Content

Vaughn VM, Yost M, Abshire C, et al. Trends in venous thromboembolism anticoagulation in patients hospitalized with COVID-19. *JAMA Netw Open.* 2021;4(6):e2111788. doi:10.1001/jamanetworkopen.2021.11788

eAppendix 1. Abstraction Tool

eAppendix 2. Definition of Sex, Race, Ethnicity

eAppendix 3. COVID Case Selection/Sampling Procedure

eFigure 1. Study Flow Diagram

eFigure 2. 60-day Mortality Over Time by Venous Thromboembolism Prophylaxis Adherence

eTable 1. IPTW Models

eTable 2. Anticoagulants Given for VTE Prophylaxis, N=1127

eTable 3. Treatment Dose Anticoagulants, N=219

eTable 4. Anticoagulant Use on Day of Death N=313

eTable 5. Cause of 60-day Mortality, N=313

eTable 6. Sensitivity Analysis When 21 Patients Who Did Not Receive Treatment Dose Anticoagulation Until After a Confirmed VTE Were Classified as Prophylactic-Dose Anticoagulation Only

eTable 7. VTE Location in Patients Who Had a 60-day VTE Event

eTable 8. VTE Timing (Out Of All Patients)

This supplemental material has been provided by the authors to give readers additional information about their work.

© 2021 Vaughn VM et al. *JAMA Network Open.*

eAppendix 1. Abstraction Tool

SELECT ALL ANTICOAGULANTS PRIOR TO THE HOSPTIAL ENCOUNTER (admission assessment)

Instructions: Review the medical record to determine the name of the anticoagulant the patient received in the 30 days prior to the hospital encounter (ER, Obs, Inpt). There are sixteen (18) options for this question.
Select all that apply:

- *"Apixaban (Eliquis)"*
- *"Argatroban"*
- *"Aspirin"*
- *"Betrixaban (Bevyxxa)"*
- *"Bivalirudin (Angiomax)"*
- *"Clopidogrel (Plavix)"*
- *"Dabigatran (Pradaxa)"*
- *"Dalteparin (Fragmin)"*
- *"Dipyridimole (platelet inhibitor)"*
- *"Edoxavan (Savaysa)"*
- *"Enoxaparin (Lovenox)"*
- *"Fonaparinux (Arixtra) 2.5 mg Daily"*
- *"Fondaparinux (Arixtra)"*
- *"Heparin"*
- *"Lepirudin (Refludon)"*
- *"Rivaroxaban (Xarelto)"*
- *"Tinzaparin (Innohep)"*
- *"Warfarin (Coumadin)"*

VENOUS THROMBOEMBOLISM (VTE) (hospitalization assessment)

Instructions: For all questions in the database, "No Answer" is the default selection. This field will be considered a blank field and is interpreted as missing data. Be sure to select the appropriate answer for each question.
Note: The term "hospital encounter" includes the timeframe from which the patient has first contact with your hospital (via ER, Observation, transfer, etc.) until they are discharged from your site.

1. WAS THE PATIENT DIAGNOSED WITH A CONFIRMED OR SUSPECTED DEEP VEIN THROMBOSIS (DVT) DURING THE HOSPITAL ENCOUNTER?

Instructions: Review the medical record to determine if the patient was diagnosed with a deep vein thrombosis (DVT) during the hospital encounter. DVT may occur in the upper extremities and/or lower extremities. There are three (3) options for this question.
INCLUDE**:** Documentation of a new diagnosis of DVT (acute or chronic) in one or more of the deep veins of the lower extremity (common iliac vein, internal iliac vein, external iliac vein, common femoral vein, deep femoral (profunda femoris) vein, femoral vein, popliteal vein, gastrocnemius vein, anterior tibial vein, soleus (soleal sinus) vein, peroneal vein, posterior tibial vein) and/or the upper extremity (subclavian vein, axillary vein, brachial vein, radial vein, ulnar vein, innominate vein [brachiocephalic]).
Documentation of a mural thrombus in one of these veins. Also include a pre-existing deep vein thrombosis (DVT) that is noted to be present on admission.
EXCLUDE**:** Superficial veins of the upper and lower extremities (cephalic vein, median cephalic vein, basilic vein, median cubital vein, median forearm vein, greater saphenous vein, lesser saphenous vein), internal/external jugular thrombosis, clots located within central venous catheters (CVCs) (even if the CVC is located within a deep vein, as long as the clot is only within the catheter, e.g. pericatheter thrombus), and hepatic/renal/splenic/mesenteric thromboses.
Select one of the following:

- *"Yes"* if the medical record indicates that the patient was diagnosed with a DVT

during the hospital encounter or a DVT is noted to be present on admission to the hospital encounter. ***Answer questions 1.1 through 1.5***
• *"No"* if the medical record does not indicate that the patient was diagnosed with a DVT during the hospital encounter and a DVT was not noted to be present on admission to the hospital encounter.
• *"Unknown"* if the medical record is silent as to whether the patient was diagnosed with a DVT during the hospital encounter or if they had a DVT present on admission to the hospital encounter.

1.1 WAS THE DEEP VEIN THROMBOSIS (DVT):
• "*Confirmed*" if the medical record indicates that the deep vein thrombosis (DVT) was confirmed via diagnostic imaging.
• "*Suspected*" if the medical record indicates that the deep vein thrombosis (DVT) was not confirmed via diagnostic imaging; however, it was highly suspicious given the clinical scenario.

1.2 DATE OF THE FIRST CONFIRMED/SUSPECTED DVT
Instructions: Review the medical record to determine the date that the DVT was diagnosed. This is likely to be found on a diagnostic study report (i.e. Doppler study, CT, etc.) or within the progress notes. Enter the date in the MM/DD/YYYY format. Use 01/01/1900 if the date is unknown.

1.3 WHAT TEST(S) WERE USED FOR THE DIAGNOSIS OF THE CONFIRMED/SUSPECTED DVT?
Instructions: Review the medical record to determine which test(s) were used to confirm the diagnosis of the DVT. If more than one test was used, check all tests that apply. There are seven (7) options for this question.
Select all that apply:
• *"Computerized Tomography Scan (CT)"* if the medical record indicates that the DVT was diagnosed via CT scan.
INCLUDE: Computerized Tomography Scan (CT), computed tomography, CAT scan, CT scan
• *"Magnetic Resonance Imaging (MRI)"* if the medical record indicates that the DVT was diagnosed via Magnetic Resonance Imaging (MRI).
• *"Ultrasound with Doppler"* if the medical record indicates that the DVT was diagnosed via ultrasound with doppler.
INCLUDE: Ultrasound with doppler, doppler ultrasound, duplex ultrasound, venous doppler ultrasound
• *"Ultrasound without Doppler"* if the medical record indicates that the DVT was diagnosed via ultrasound without doppler.
INCLUDE: ultrasound, compression ultrasound.
• *"Venogram"* if the medical record indicates that the DVT was diagnosed via venogram.
INCLUDE: venogram, phlebography
• "*None of the above*" if the medical record indicates that none of the above methods were used to diagnose the DVT.
• "*Unknown*" if the medical record is silent as to the test used for the diagnosis of the DVT.

1.4 INDICATE THE LOCATION OF THE CONFIRMED/SUSPECTED DVT
Instructions: Review the medical record to determine the location of the DVT. If more than one location is involved, check all that apply. There are five (5) options for this question.
Select all that apply:
• *"Right Lower Extremity"* if the medical record indicates that the DVT was located in one or more of the deep veins of the right lower extremity.
INCLUDE: Only include lower extremity DVTs in the right common iliac vein, internal iliac vein, external iliac vein, common femoral vein, deep femoral (profunda femoris) vein, femoral vein, popliteal vein, gastrocnemius vein, anterior tibial vein, soleus (soleal sinus) vein, peroneal vein, and/or posterior tibial vein. ***Answer question 1.4.1***
• *"Left Lower Extremity"* if the medical record indicates that the DVT was located in one or more of the deep veins of the left lower extremity.
INCLUDE: Only include lower extremity DVTs in the left common iliac vein, internal iliac vein, external iliac vein, common femoral vein, deep femoral (profunda femoris) vein, femoral vein, popliteal vein, gastrocnemius vein, anterior tibial vein, soleus (soleal sinus) vein, peroneal vein, and/or posterior

© 2021 Vaughn VM et al. *JAMA Network Open.*

tibial vein. ***Answer question 1.4.1***
• *"Right Upper Extremity"* if the medical record indicates that the DVT was located in one or more of the deep veins of the right upper extremity.
INCLUDE: Only include upper extremity DVTs in the right subclavian vein, axillary vein, brachial vein, radial vein, ulnar veins, and/or innominate (brachiocephalic). ***Answer question 1.4.2***
• *"Left Upper Extremity"* if the medical record indicates that the DVT was located in one or more of the deep veins of the left upper extremity.
INCLUDE: Only include upper extremity DVTs in the left subclavian vein, axillary vein, brachial vein, radial vein, ulnar veins, and/or innominate vein (brachiocephalic). ***Answer question 1.4.2***
• *"Unknown"* if the medical record is silent as to the location of the DVT.

1.4.1. FOR LOWER EXTREMITIES, INDICATE THE VEIN(S) INVOLVED
Instructions: Review the medical record to determine the vein in which the DVT was located. If more than one DVT was found, check all that apply. There are thirteen (13) options for this question.
Select all that apply:
• *"Common iliac"* if the medical record indicates that the DVT of the lower extremity was within in the common iliac vein.
• *"Internal iliac"* if the medical record indicates that the DVT of the lower extremity was within the internal iliac vein.
• *"External iliac"* if the medical record indicates that the DVT of the lower extremity was within the external iliac vein.
• *"Common femoral"* if the medical record indicates that the DVT of the lower extremity was within the common femoral vein.
• *"Deep femoral"* if the medical record indicates that the DVT of the lower extremity was within the deep femoral vein.
• *"Femoral"* if the medical record indicates that the DVT of the lower extremity was within the femoral vein.
• *"Popliteal"* if the medical record indicates that the DVT of the lower extremity was within the popliteal vein.
• *"Gastrocnemius"* if the medical record indicates that the DVT of the lower extremity was within the gastrocnemius vein.
• *"Anterior Tibial"* if the medical record indicates that the DVT of the lower extremity was within the anterior tibial vein.
• *"Soleus"* if the medical record indicates that the DVT of the lower extremity was within the soleus vein.
• *"Peroneal"* if the medical record indicates that the DVT of the lower extremity was within the peroneal vein.
• *"Posterior Tibial"* if the medical record indicates that the DVT of the lower extremity was within the posterior tibial vein.
• *"Unknown"* if the medical record was silent as to the vein which the lower extremity DVT was in.

1.4.2. FOR UPPER EXTREMITIES, INDICATE THE VEIN(S) INVOLVED
Instructions: Review the medical record to determine the upper extremity vein in which the DVT was located. If more than one DVT was found, check all that apply. There are seven (7) options for this question.
Select all that apply:
• *"Subclavian"* if the medical record indicates that the DVT of the upper extremity was within the subclavian vein.
• *"Brachial"* if the medical record indicates that the DVT of the upper extremity was within the brachial vein.
• *"Axillary"* if the medical record indicates that the DVT of the upper extremity was within the axillary vein.
• *"Ulnar"* if the medical record indicates that the DVT of the upper extremity was within the ulnar vein.
• *"Innominate"* if the medical record indicates that the DVT of the upper extremity was within the innominate vein.
INCLUDE: Innominate vein, brachiocephalic vein
• *"Radial"* if the medical record indicates that the DVT of the upper extremity was within the radial vein.
• *"Unknown"* if the medical record was silent as to the vein which the upper extremity DVT was in.

1.5 INDICATE THE CLASSIFICATION OF DVT FROM THE TEST RESULT
Instructions: Review the medical record to determine the classification of the DVT as reported per the test result (i.e. CT result, doppler result, etc.). If more than one DVT was found, the patient may have more than one classification of DVT, so you should select all options that apply. For example, a patient may have a chronic DVT in a lower extremity with the presence of an additional acute clot somewhere else.
There are three (3) options for this question.

© 2021 Vaughn VM et al. *JAMA Network Open.*

Select all that apply:
• *"Acute"* if the medical record indicates that the DVT was acute.
• *"Chronic"* if the medical record indicates that the DVT was chronic.
• *"Unknown"* if the medical record is silent as to whether the DVT was acute or chronic.

2. WAS THE PATIENT DIAGNOSED WITH A CONFIRMED OR SUSPECTED PULMONARY EMBOLISM (PE) DURING THE HOSPITAL ENCOUNTER?
Instructions: Review the medical record to determine if the patient was diagnosed with a pulmonary embolism (PE) during the hospital encounter. There are three (3) options for this question.
INCLUDE: A new pulmonary embolism (PE) diagnosed during the hospital encounter, also include a pre-existing pulmonary embolism that is present on admission.
EXCLUDE: Non blood clot emboli such as septic pulmonary emboli.
Select one of the following:
• *"Yes"* if the medical record indicates that the patient was diagnosed with a PE during the hospital encounter or a PE was present on admission to the hospital encounter.
Answer questions 2.1 through 2.3
• *"No"* if the medical record does not indicate that the patient was diagnosed with a PE during the hospital encounter or a PE was present on admission to the hospital encounter.
• *"Unknown"* if the medical record is silent as to whether or not the patient was diagnosed with a PE during the hospital encounter or a PE was present on admission to the hospital encounter.

2.1 WAS THE PULMONARY EMBOLISM (PE):
• "*Confirmed*" if the medical record indicates that the pulmonary embolism (PE) was confirmed via diagnostic imaging.
• "*Suspected*" if the medical record indicates that the pulmonary embolism (PE) was not confirmed via diagnostic imaging; however, a PE is highly suspicious given the clinical scenario.

2.2 DATE OF THE FIRST CONFIRMED/SUSPECTED PE
Instructions: Review the medical record to determine the date that the pulmonary embolism (PE) was diagnosed. This is likely to be found on a diagnostic study report (i.e. CT, MRI, etc.) or within the progress notes. Enter the date in the MM/DD/YYYY format. Use 01/01/1900 if the date is unknown.

2.3 WHAT TEST(S) WERE USED FOR THE DIAGNOSIS OF THE CONFIRMED/SUSPECTED PE?
Instructions: Review the medical record to determine which test(s) were used to confirm the diagnosis of the PE. If more than one test was used, check all tests that apply. There are six (6) options for this question.
Select all that apply:
• *"Computerized Tomography Scan (CT)"* if the medical record indicates that the PE was diagnosed via CT scan.
INCLUDE: Computerized Tomography Scan (CT), computed tomography, CAT scan, CT scan
• *"Magnetic Resonance Imaging (MRI)"* if the medical record indicates that the PE was diagnosed via Magnetic Resonance Imaging (MRI).
• *"Pulmonary Venogram"* if the medical record indicates that the PE was diagnosed via pulmonary venogram.
INCLUDE: Pulmonary venogram, pulmonary angiography
• *"Ventilation/Perfusion scan"* if the medical record indicates that the PE was diagnosed via ventilation/perfusion scan.
INCLUDE: Ventilation/perfusion scan, V/Q scan
• *"Other"* if the medical record indicates that the PE was diagnosed via a test not indicated above.
• "*None of the above*" if the medical record indicates that none of the methods above were used to diagnose the PE.
• "*Unknown*" if the medical record is silent as to the test used for the diagnosis of the PE.

ANTICOAGULANTS (daily assessment)
Instructions: For all questions in the database, "No Answer" is the default selection. This field will be considered a blank field and is interpreted as missing data. Be sure to select the appropriate answer for each question.
Note: All of the following questions will populate for data collection on all days of the hospital encounter.

© 2021 Vaughn VM et al. *JAMA Network Open.*

1. WAS A TREATMENT ANTICOAGULANT ADMINISTERED ON THE DATE INDICATED ABOVE?

Instructions: Review the medical record to determine if the patient received a treatment anticoagulant on the date indicated. List of treatment anticoagulants and dosages listed under 1.1.

Note: Only enter anticoagulants that were administered to the patient (at least one dose). If the anticoagulant was ordered, but the patient refused all doses on the selected date, do not enter for the selected date.

Select one of the following:

• *"Yes"* if there is documentation that a treatment anticoagulant was administered to the patient on the date indicated above. ***Answer question 1.1***

• *"No"* if there is no documentation that a treatment anticoagulant was administered on the date indicated above.

• "*Unknown*" select if the medical record is silent as to whether the patient received a treatment anticoagulant on the date indicated above.

1.1 SELECT ALL TREATMENT ANTICOAGULANTS ADMINISTERED ON THE DATE INDICATED ABOVE.

Instructions: Review the medical record to determine all of the treatment anticoagulants administered to the patient on the date indicated above. Select all that apply:

• *"Apixaban (Eliquis)"*

• *"Argatroban"*

• *"Bivalirudin (Angiomax)"*

• *"Dabigatran (Pradaxa)"*

• *"Dalteparin (Fragmin)"*

Note: TREATMENT DOSE EXAMPLE: For unstable angina/non Qwave MI the recommended dose is 120 international units/kg; for a patient weighing 150 pounds give 8,182 international units) q 12 hours' x 58 days, maximum 10,000 international units, q 12 hours' x 58 days with concurrent aspirin.

• *"Edoxavan (Savaysa)"*

• *"Enoxaparin (Lovenox)"*

Note: TREATMENT DOSE EXAMPLE: Unstable angina and nonQwave MI: 1mg/kg (68 mg for 150# person) SQ every 12 hours with aspirin

• *"Fondaparinux (Arixtra)"*

Note: TREATMENT DOSING EXAMPLE: Deep vein thrombosis/PE: Adults
<50kg: 5 mg SQ daily; 50100kg: 7.5mg SC daily; and >100kg: 10 mg SQ daily

• *"Heparin (Intravenous)"*

• *"Lepirudin (Refludon)"*

• *"Rivaroxaban (Xarelto)"*

Note: TREATMENT DOSING EXAMPLE: 20 mg PO Daily for Afib. For Creatinine Clearance between 1550ml/min, 15 mg Daily is recommended.

• *"Tinzaparin (Innohep)"*

• *"Warfarin (Coumadin)"*

2. WAS AN ANTICOAGULANT ADMINISTERED FOR VTE PROPHYLAXIS ON THE DATE INDICATED ABOVE?

Instructions: Review the medical record to determine if the patient received an anticoagulant for VTE prophylaxis on the date indicated. List of prophylactic anticoagulants and dosages listed under 2.1.

Note: Only enter anticoagulants that were administered to the patient (at least one dose). If the anticoagulant was ordered, but that patient refused all doses on the selected date, do not enter for the selected date.

Select one of the following:

• *"Yes"* if there is documentation that an anticoagulant for VTE prophylaxis was administered on the date indicated above. ***Answer question 2.1***

• *"No"* if there is no documentation that an anticoagulant for VTE prophylaxis was administered on the date indicated above.

• "*Unknown*" select if the medical record is silent as to whether the patient received an anticoagulant for VTE prophylaxis on the date indicated above.

2.1 SELECT ALL VTE PROPHYLAXIS ANTICOAGULANTS ADMINISTERED ON THE DATE INDICATED ABOVE.

© 2021 Vaughn VM et al. *JAMA Network Open.*

Instructions: Review the medical record to determine all of the VTE prophylaxis anticoagulants administered to the patient on the date indicated above.
Select all that apply:

- “*Apixaban*”
- *“Aspirin”*
- *“Argatroban”*
- *“Bextrixaban (Bevyxxa) 40 mg Daily”*
- *“Betrixaban (Bevyxxa) 80mg Daily”*
- *“Clopidogrel (Plavix)”*
- *“Dabigatran”*
- *“Dalteparin (Fragmin) 5,000 Units Daily”*
- *“Dipyridimole (platelet inhibitor)”*
- *“Enoxaparin (Lovenox) 30 mg BID”*
- *“Enoxaparin (Lovenox) 30 mg Daily (for CrCl <30)”*
- *“Enoxaparin (Lovenox) 40 mg BID (for obese patients)”*
- *“Enoxaparin (Lovenox) 40 mg Daily”*
- *“Fondaparinux (Arixtra) 2.5 mg Daily”*
- *“Heparin (Intravenous)”*
- *“Heparin 5,000 Units BID”*
- *“Heparin 5,000 Units TID”*
- *“Heparin 7,500 Units TID (for morbid obesity)”*

Note: Criteria of BMI >= 30 OR Documentation of ‘Obese’ when BMI is unavailable

- *“Rivaroxaban (Xarelto)”*

© 2021 Vaughn VM et al. *JAMA Network Open.*

eAppendix 2. Definition of Sex, Race, Ethnicity

GENDER
Instructions: Review the medical record to determine the gender of the patient.
This is a required field and the form cannot be submitted without an entry in this field.
Select one of the following:

- *"Male"* if the patient is categorized as a man in the medical record.
- *"Female"* if the patient is categorized as a woman in the medical record.
- *"Unknown"* if the patient's gender is unknown.

ETHNICITY
Instructions: Review the medical record to determine the patient's ethnicity.
Select one of the following:

- *"Hispanic or Latino"* if patient demographic information indicates patient is of Hispanic descent. The US Census Bureau states that "People who identify their origin as Spanish, Hispanic, or Latino may be of any race."
- *"Non-Hispanic or Latino"* if patient demographic information indicates patient is not of Hispanic descent.
- "*Unknown*" if ethnicity is not reported in the medical record.

RACE
Instructions: Review the medical record to determine the patient's race.
Select one of the following:

- *"American Indian or Alaskan Native"* if patient demographic information indicates patient is Native American, American Indian, or Alaska Native.
- *"Arab and Chaldean Ancestries"* if the patient demographic information indicate patient is of Arab or Chaldean Ancestries.
- *"Asian"* if patient demographic information indicates Asian.
- *"Black or African American"* if patient demographic information indicates patient is black or African American.
- *"Native Hawaiian or Pacific Islander"* if patient demographic information indicates patient is Native Hawaiian or Pacific Islander.
- *"White or Caucasian"* if patient demographic information indicates patient is white or Caucasian.
- *"Other"* if patient demographic information indicates the patient is a race other than what is listed above.
- *"Unknown"* if patient's race is not indicated in the medical record.

© 2021 Vaughn VM et al. *JAMA Network Open.*

eAppendix 3. COVID Case Selection/Sampling Procedure

Data collection for the Mi-COVID 19 Initiative will involve the abstraction of patient data for as many eligible cases as possible for hospitals who volunteer to participate.

The clinical data abstractor will review potential cases and enter basic demographic and testing information on as many COVID-19 cases as is possible. Then, a subset of eligible cases will be randomized for detailed data entry (full abstraction). The number of cases randomized for demographicentry and full abstraction is dependent on the volume of eligible cases and the availability of data abstraction personnel within the participating hospital. As this is volunteer participation, there is no minimum case volume requirement.

A random sampling strategy will be utilized to select cases for demographic entry, and full abstraction atsites where the volume of eligible cases is high and/or data abstraction resources are limited. At sites where the volume of eligible cases is low and sufficient data abstraction resources are available, complete full abstraction of all eligible cases.

The random sampling strategy for selection of cases for full abstraction will be followed in order to establish a sample and to minimize sampling bias.

1. This process involves first selecting eligible cases from each day of a cycle, if eligible cases are available.
2. For each day with eligible cases, review the discharge date timestamp [hh:mm] from the hospital encounter (Admission or Observation, for observation only cases).
3. From the timestamp [hh:mm] review, select the first eligible case with the smallest *minute* value. For example, if a site has 4 eligible COVID-19 cases discharged on the same day at thesetimes: 9:17, 10:27, 12:16 & 21:01, who would qualify for the first eligible case? The patient discharged at 21:01 qualifies as the first case for full abstraction for this day, as :01 is the smallest minute value from the list of discharge times of eligible cases on this day. Repeat thissame process for selecting the second, third, and subsequent cases for this day. The goal is to have a least one case from each day of the week if available.

Example for selecting a patient sample:

Discharge Day:	**Monday**	**Tuesday**	**Wednesday**	**Thursday**	**Friday**	**Saturday**	**Sunday**
Initial Cases	Patient 1: Discharged at 20:07	Patient 2: Discharged at 17:03	Patient 3: Discharged at 09:01	Patient 4: Discharged at 14:10	Patient 5: Discharged at 09:02	Patient 6: Discharged at 11:13	Patient 7: Discharged at 10:25
Additional Cases (if available)	Patient 8: Discharged at 13:14		Patient 9: Discharged at 08:25	Patient 10: Discharged at 14:45		Patient 11: Discharged at 09:55	

NOTE 1: Hospital encounter includes emergency, observation, and inpatient (for admissions) time in the hospital.
NOTE 2: Hospital discharge includes patients discharged alive from inpatient or observation, and patients discharged due to death.
NOTE 3: In the event that the smallest minute value on more than one case is the same, select the casewhere the patient is discharged later in the day. (For example discharge timestamps of 12:10 and 13:10are both part of the sample. Select the case where the patient was discharged at 13:10).

Eligible cases will include the following:

- Discharged from hospitalization or observation that includes 1 **<u>or</u>** more of the following:
  - COVID-19 or SARS-CoV-2 testing collected and resulted as positive during the hospital

© 2021 Vaughn VM et al. *JAMA Network Open.*

encounter OR in the 21 days prior to the hospital encounter.
- COVID-19 or SARS-CoV-2 testing collected and resulted as negative during the hospital encounter OR in the 21 days prior to the hospital encounter with one or more of the following symptoms noted in the Emergency Room Note, Observation Note and/or History & Physical: Cough, Dyspnea (shortness of breath) or Fever (>100.4 [F]). If the testing was completed during the hospital encounter the above symptom must be noted in the progress notes on the day prior to testing, day of testing, or the day after testing.
- Discharge Diagnosis of ICD-10 for COVID: U07.1, 2019-nCoV acute respiratory disease.
- Strong clinical suspicion of COVID-19 infection that is documented but is unable to be confirmed or coded due to logistical constraints.
  - Example ICD-10 Codes:
    - B34.2 - Coronavirus infection, unspecified
    - B97.21 - SARS-associated coronavirus infection
    - B97.29 - Other coronavirus as the cause of diseases classified elsewhere
    - Z20.828 – Contact with and (suspected) exposure to other viral communicable diseases

- Patients did not meet any of the following exclusions:
  - Pregnant
  - Under the age of 18
  - Left against medical advice (AMA)
  - Comfort Care/Hospice within 3 hours of the hospital encounter
  - Length of stay greater than 120 days during index encounter
  - Patient discharge falls within the 60-day follow up window of a previously recorded/abstracted admission

The process of filtering out non-eligible cases may happen electronically or manually.

COVID Positive or Negative Abstraction Ratio

Abstraction of COVID positive cases and those with clinical suspicion/presumed COVID19, however COVID testing resulted as negative, are 2 key groups targeted for abstraction. To ensure an appropriate sample of both types, we ask that abstraction (both for demographics only and full abstractions) be completed at a 2:1 ratio for positive to negative COVID19 cases (i.e. for every 2 COVID positive abstractions, 1 COVID negative plus symptom(s) abstraction be completed). Please see above criteria for eligibility requirements for patients who are positive vs. negative. This applies to all hospitals whether they complete a full abstraction for all cases or a subset of cases.

Overall Abstraction Process

The data collection for each eligible case will capture data from the hospitalization of interest (ER, Observation, Admission) through 60 days post-hospitalization. This is a two phase abstraction process.

To accomplish the selection of cases, the clinical data abstractor should do the following:

1) Obtain a list of patients based on the specifications above. Note: these patients can be from all services (i.e. medicine, surgery, etc.) during the timeframe covered by the cycle. The list may or maynot already have exclusion filters applied depending on the capabilities of the hospital. Please note that the list can be pulled in multiple ways:
   a. Laboratory testing
   b. Discharge ICD 10 code
   c. Site specific tracking of patients who have been determined to have a COVID infection. The reason we allow several methods is to allow flexibility given the capabilities of different medical record systems. It is not necessary to obtain cases through all methods. Please utilize the most reliablesource of information available.

2) For sites completing full abstraction on **all** cases, complete the following steps:
   a. Determine patient eligibility
      i. If ineligible (excluded), log this patient as excluded on your patient list
   b. If eligible (included), enroll the patient into the database and complete full data abstractionin two phases
      i. Phase 1: Initial Hospitalization
      ii. Phase 2: 60 Day Review
3) For sites completing full abstraction on a **subset** of cases, complete the following steps:
   a. Determine patient eligibility
      i. If ineligible (excluded), log this patient as excluded on your patient list
   b. If eligible (included), enroll the patient into the database and complete basic demographicand testing information
   c. To determine which cases require full abstraction, complete the following steps:
      i. Organize the list of eligible cases chronologically by date and time of discharge
      ii. Begin with the first date in the cycle, with eligible cases.
      iii. Review eligible cases within this first date for the patient whose timestamp minute value is the smallest. This case qualifies for full abstraction.
      iv. Complete full data abstraction in two phases
         1. Phase 1: Initial Hospitalization
         2. Phase 2: 60 Day Review
      v. Repeat this process by continuing to review patients from the chronological list making sure that cases are distributed across all the days of the week to the greatest extent possible (meaning discharge dates across all days of the week Sunday-Saturday).

© 2021 Vaughn VM et al. *JAMA Network Open.*

eFigure 1. Study Flow Diagram

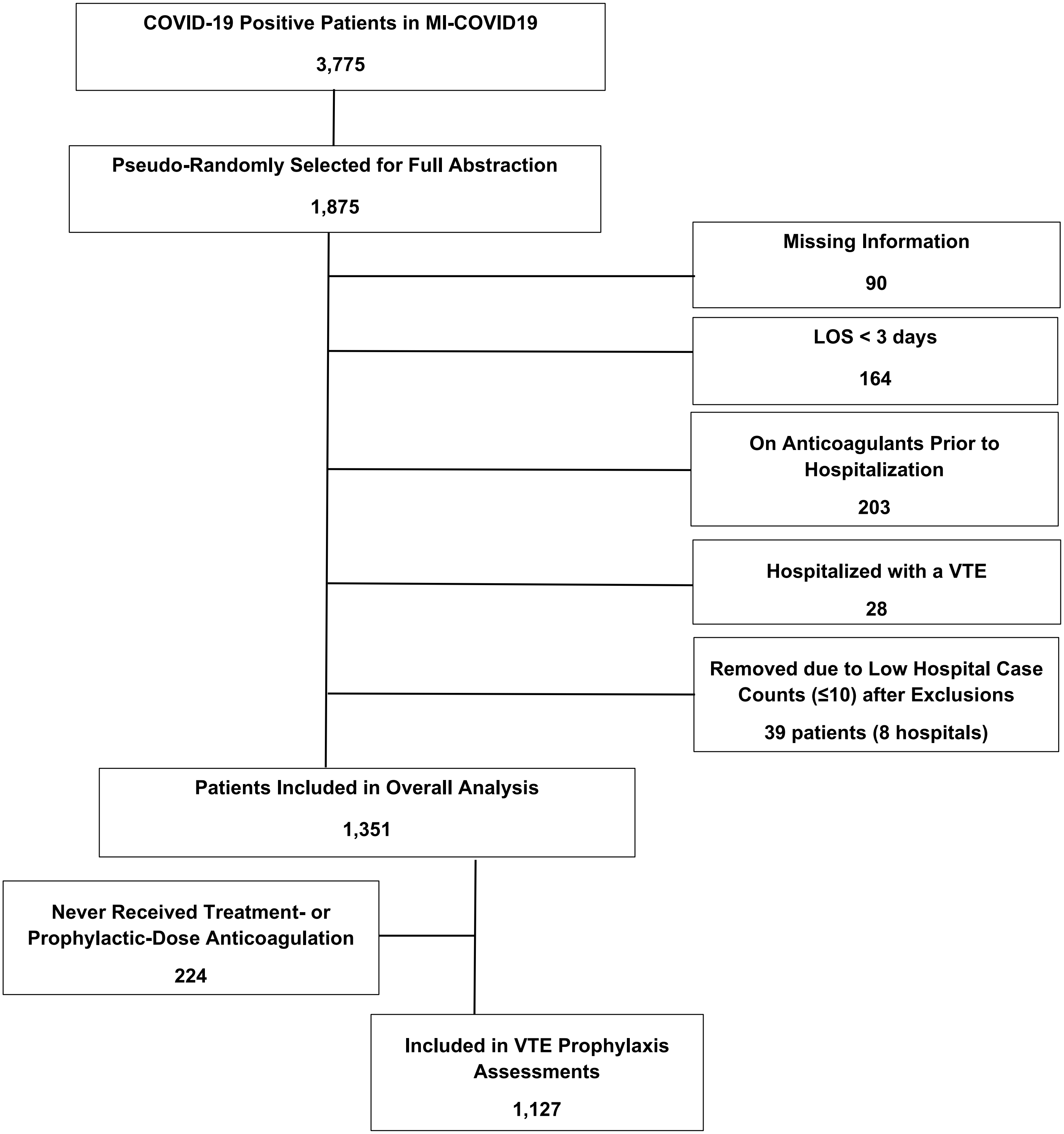

© 2021 Vaughn VM et al. *JAMA Network Open.*

eFigure 2. 60-day Mortality Over Time By Venous Thromboembolism Prophylaxis Adherence

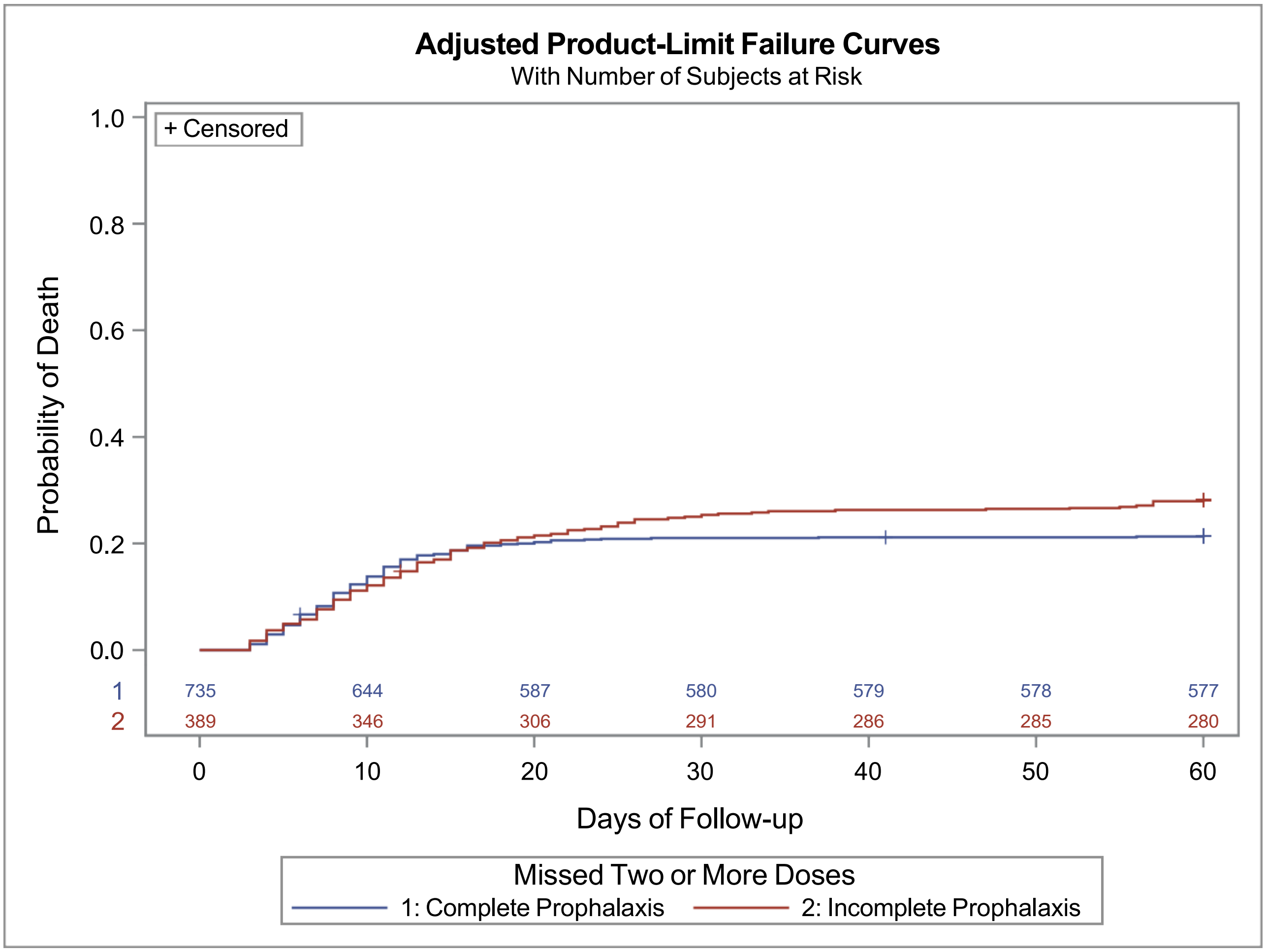

Adjusted hazard models showing the hazard of death up to 60-days after hospitalization. Each patient was weighted by the inverse probability of being in their anticoagulant exposure group. Only patients who ever received treatment or prophylactic-dose anticoagulation are included. The two exposure groups are: 1) VTE prophylaxis adherent (i.e., missing <2 days of pharmacologic venous thromboembolism prophylaxis) and 2) VTE prophylaxis non-adherent (i.e., missing 2 or more days of pharmacologic venous thromboembolism prophylaxis). After adjustment, there was a significant difference in 60-day mortality between those with vs. without non-adherence (aHR=1.312 [95% CI: 1.028, 1.674]).

© 2021 Vaughn VM et al. *JAMA Network Open.*

eTable 1. IPTW Models

A. Anticoagulant Groups

	Unweighted					Weighted				
	**No Anti-coagulation**	**Prophylactic-dose Only**	**Ever Treatment-Dose**	**ST Diff No-Prophy**	**ST Diff No-Treat**	**No Anti-coagulation**	**Prophylactic-dose Only**	**Ever Treatment-Dose**	**ST Diff No-Prophy**	**ST Diff No-Treat**
Age	57.4±17.9	64±16.2	66.5±13.9	0.02	0.01	65.6±17.8	63.8±16.2	65.7±15.4	0.01	0.00
Week of Discharge	3.4±1.7	3.4±2.1	5.8±2.5	0.00	0.45	4.6±2.4	4.6±2.2	4.6±2.1	0.00	0.00
**Ethnicity**										
Nonhispanic	136 (84%)	824 (84.9%)	197 (90%)	-0.03	-0.18	136 (87.4%)	828 (85.7%)	188 (86.9%)	0.05	-0.03
Hispanic	10 (6.2%)	53 (5.5%)	9 (4.1%)	0.03	0.09	5 (3.4%)	50 (5.2%)	10 (4.5%)	-0.09	0.03
Unknown	16 (9.9%)	93 (9.6%)	13 (5.9%)	0.01	0.15	14 (9.2%)	88 (9.1%)	19 (8.6%)	0.00	0.02
**Race**										
Black	80 (49.4%)	475 (49%)	106 (48.4%)	0.01	0.02	70 (44.9%)	478 (49.5%)	102 (47.2%)	-0.09	0.05
White	67 (41.4%)	384 (39.6%)	89 (40.6%)	0.04	0.01	72 (46.3%)	379 (39.2%)	94 (43.5%)	0.14	-0.09
Asian	6 (3.7%)	26 (2.7%)	4 (1.8%)	0.06	0.12	3 (2.2%)	26 (2.7%)	7 (3.3%)	-0.03	-0.04
Other	4 (2.5%)	36 (3.7%)	16 (7.3%)	-0.07	-0.23	6 (4%)	42 (4.4%)	8 (3.5%)	-0.02	0.05
Unknown	5 (3.1%)	49 (5.1%)	4 (1.8%)	-0.10	0.08	4 (2.5%)	42 (4.3%)	5 (2.5%)	-0.10	0.10
**Respiratory Support**										
Mechanical ventilation	9 (5.6%)	118 (12.2%)	93 (42.5%)	-0.24	-0.94	24 (15.1%)	155 (16%)	34 (15.8%)	-0.02	0.01
Non-invasive mechanical ventilation	0 (0%)	12 (1.2%)	6 (2.7%)	-0.22	-0.33	0 (0%)	13 (1.3%)	3 (1.3%)	-0.23	0.00
Heated high-flow nasal cannula	5 (3.1%)	70 (7.2%)	18 (8.2%)	-0.19	-0.23	8 (5.2%)	70 (7.3%)	17 (7.9%)	-0.09	-0.02
Low flow oxygen	86 (53.1%)	563 (58%)	81 (37%)	-0.10	0.33	83 (53.3%)	522 (54.1%)	119 (54.8%)	-0.02	-0.01
No supplemental oxygen	62 (38.3%)	207 (21.3%)	21 (9.6%)	0.37	0.70	41 (26.3%)	206 (21.3%)	44 (20.2%)	0.12	0.03

© 2021 Vaughn VM et al. *JAMA Network Open.*

Female	89 (54.9%)	469 (48.4%)	87 (39.7%)	0.13	0.31	79 (50.6%)	460 (47.7%)	99 (45.8%)	0.06	0.04

	Unweighted					Weighted				
	**No Anti-coagulation**	**Prophylactic-dose Only**	**Ever Treatment-Dose**	**ST Diff No-Prophy**	**ST Diff No-Treat**	**No Anti-coagulation**	**Prophylactic-dose Only**	**Ever Treatment-Dose**	**ST Diff No-Prophy**	**ST Diff No-Treat**
**BMI**										
No Data	8 (4.9%)	56 (5.8%)	9 (4.1%)	-0.04	0.04	8 (4.9%)	52 (5.4%)	11 (5.1%)	-0.02	0.01
≤25	36 (22.2%)	199 (20.5%)	37 (16.9%)	0.04	0.13	31 (20.1%)	196 (20.2%)	51 (23.4%)	0.00	-0.08
<30	36 (22.2%)	285 (29.4%)	53 (24.2%)	-0.16	-0.05	42 (26.8%)	270 (27.9%)	56 (26%)	-0.02	0.04
≥30	82 (50.6%)	430 (44.3%)	120 (54.8%)	0.13	-0.08	75 (48.2%)	449 (46.5%)	98 (45.5%)	0.03	0.02
Hypertension	92 (56.8%)	653 (67.3%)	157 (71.7%)	-0.22	-0.31	112 (72%)	647 (66.9%)	150 (69.1%)	0.11	-0.05
Chronic Kidney Disease	25 (15.4%)	266 (27.4%)	68 (31.1%)	-0.29	-0.37	54 (34.6%)	257 (26.6%)	58 (26.9%)	0.17	-0.01
Congestive heart failure	17 (10.5%)	110 (11.3%)	38 (17.4%)	-0.03	-0.20	23 (15%)	120 (12.5%)	28 (13.1%)	0.08	-0.02
**D-dimer, xULN**										
No data	122 (75.3%)	572 (59%)	101 (46.1%)	0.35	0.61	103 (65.9%)	570 (59%)	116 (53.5%)	0.14	0.11
≤2	25 (15.4%)	224 (23.1%)	53 (24.2%)	-0.20	-0.22	31 (19.7%)	217 (22.5%)	50 (23.1%)	-0.07	-0.01
≤4	6 (3.7%)	99 (10.2%)	24 (11%)	-0.26	-0.29	8 (5%)	91 (9.4%)	25 (11.4%)	-0.18	-0.07
>4	9 (5.6%)	75 (7.7%)	41 (18.7%)	-0.09	-0.42	15 (9.4%)	87 (9%)	26 (12%)	0.01	-0.10
Remdesivir	0 (0%)	20 (2.1%)	5 (2.3%)	-0.29	-0.30	0 (0%)	17 (1.7%)	2 (0.9%)	-0.26	0.08
Corticosteroids	19 (11.7%)	247 (25.5%)	111 (50.7%)	-0.36	-0.89	40 (25.8%)	272 (28.2%)	56 (25.8%)	-0.05	0.05
IL-6 receptor inhibitor	1 (0.6%)	21 (2.2%)	22 (10%)	-0.14	-0.49	1 (0.6%)	31 (3.2%)	8 (3.7%)	-0.20	-0.03

B. IPTW Models- For Prophylaxis Adherent vs. Non-Adherent Groups

	Unweighted			Weighted		
	**Adherent**	**Non-adherent**	**Standardized Difference**	**Adherent**	**Non-adherent**	**Standardized Difference**
Age	63.7±15.4	65.3±16.5	0.01	64.2±15.5	64.3±16.6	0.00
Week of Admission	3.9±2.3	3.3±1.7	0.15	3.7±2.2	3.7±2.1	0.00
Ethnicity						
Nonhispanic	631 (85.9%)	334 (85.2%)	0.02	629 (85.4%)	332 (85.3%)	0.00
Hispanic	45 (6.1%)	17 (4.3%)	0.08	41 (5.6%)	22 (5.7%)	-0.01

© 2021 Vaughn VM et al. *JAMA Network Open.*

Unknown	59 (8%)	41 (10.5%)	-0.08	66 (9%)	35 (9%)	0.00
**Race**						
Black	374 (50.9%)	181 (46.2%)	0.09	363 (49.4%)	192 (49.4%)	0.00
White	273 (37.1%)	174 (44.4%)	-0.15	293 (39.8%)	155 (39.8%)	0.00
Asian	14 (1.9%)	14 (3.6%)	-0.10	17 (2.3%)	9 (2.4%)	-0.01
Other	34 (4.6%)	13 (3.3%)	0.07	30 (4.1%)	15 (3.9%)	0.01
Unknown	40 (5.4%)	10 (2.6%)	0.15	33 (4.4%)	17 (4.5%)	0.00
**Respiratory Support**						
Mechanical ventilation	114 (15.5%)	81 (20.7%)	-0.13	132 (17.9%)	70 (18%)	0.00
Non-invasive mechanical ventilation	11 (1.5%)	7 (1.8%)	-0.02	12 (1.7%)	9 (2.4%)	-0.05
Heated high-flow nasal cannula	45 (6.1%)	40 (10.2%)	-0.15	53 (7.2%)	29 (7.4%)	-0.01
Low flow oxygen	410 (55.8%)	204 (52%)	0.08	399 (54.2%)	207 (53.2%)	0.02
No supplemental oxygen	155 (21.1%)	60 (15.3%)	0.15	140 (19%)	74 (19.1%)	0.00
Female	336 (45.7%)	192 (49%)	-0.07	342 (46.5%)	181 (46.6%)	0.00
**BMI**						
No Data	30 (4.1%)	30 (7.7%)	-0.15	43 (5.8%)	22 (5.6%)	0.01
≤25	140 (19%)	85 (21.7%)	-0.07	148 (20.2%)	70 (18%)	0.06
<30	196 (26.7%)	130 (33.2%)	-0.14	196 (26.6%)	130 (33.3%)	-0.15
≥30	369 (50.2%)	147 (37.5%)	0.26	349 (47.4%)	168 (43.1%)	0.09
Kidney Disease	204 (27.8%)	112 (28.6%)	-0.02	205 (27.9%)	108 (27.7%)	0.00
CHF	87 (11.8%)	46 (11.7%)	0.00	85 (11.6%)	45 (11.6%)	0.00
**D-dimer, xULN**						
No data	417 (56.7%)	236 (60.2%)	-0.07	430 (58.5%)	229 (58.8%)	-0.01
≤2	164 (22.3%)	91 (23.2%)	-0.02	165 (22.4%)	87 (22.4%)	0.00
≤4	83 (11.3%)	34 (8.7%)	0.09	75 (10.2%)	40 (10.3%)	0.00
>4	71 (9.7%)	31 (7.9%)	0.06	65 (8.9%)	33 (8.6%)	0.01
Remdesivir	9 (1.2%)	15 (3.8%)	-0.17	15 (2%)	8 (2.1%)	-0.01
Corticosteroids	9 (1.2%)	15 (3.8%)	-0.17	215 (29.3%)	109 (28%)	0.03

© 2021 Vaughn VM et al. *JAMA Network Open.*

IL-6 receptor inhibitor	208 (28.3%)	122 (31.1%)	-0.06	26 (3.6%)	14 (3.6%)	0.00
Treatment Prophylactic Anticoagulants	22 (3%)	18 (4.6%)	-0.08	103 (14%)	52 (13.4%)	0.02

© 2021 Vaughn VM et al. *JAMA Network Open.*

eTable 2. Anticoagulants Given for VTE Prophylaxis, N=1127

	**ICU (N=360)**<br>**N (%)**	**Non-ICU (N=767)**<br>**N (%)**
Enoxaparin (Lovenox) 30-40 mg daily	200 (55.6%)	470 (61.3%)
Enoxaparin (Lovenox) 30-40 mg BID	59 (16.4%)	80 (10.4%)
Fondaparinux (Arixtra) 2.5 mg Daily	3 (0.8%)	19 (2.5%)
Heparin ≤15,000 daily units	172 (47.8%)	290 (37.8%)
Heparin >15,000 daily units	12 (3.3%)	3 (0.4%)
Apixaban (with prophylactic intent)	4/257 (1.6%)	2/537 (0.4%)
Intravenous Heparin (with prophylactic intent)	5/257 (2.0%)	4/537 (0.7%)

Numbers may add up to >100% if patients received multiple different anticoagulants. The following anticoagulants were added on 5/2: Apixaban, Heparin (IV), Rivaroxaban. No patients were documented as receiving Betrixaban or Rivaroxaban as prophylaxis.

© 2021 Vaughn VM et al. *JAMA Network Open.*

eTable 3. Treatment Dose Anticoagulants, N=219

	ICU (N=131) N (%)	Non-ICU (N=102) N (%)	P-value
Apixaban (Eliquis)	16 (12.2%)	25 (24.5%)	0.015
Edoxavan (Savaysa)	0	1 (1.0%)	0.256
Enoxaparin (Lovenox)	64 (48.9%)	59 (57.8%)	0.173
Heparin (Intravenous)	83 (63.4%)	29 (28.4%)	<.001
Rivaroxaban (Xarelto)	4 (3.1%)	3 (2.9%)	0.960
Warfarin (Coumadin)	1 (0.8%)	4 (3.9%)	0.099

Numbers may add up to >100% if patients received multiple different anticoagulants.

© 2021 Vaughn VM et al. *JAMA Network Open.*

eTable 4. Anticoagulant Use on Day of Death N=313

	**Anticoagulant Exposure Categorization**		
**Anticoagulant Exposure on Day of Death**	**Deaths in No Anticoagulation Group (N=23); N (%)**	**Deaths in Prophylactic Only Group (N=203); N (%)**	**Death in Ever Treatment Dose Anticoagulants Group (N=87); N (%)**
No anticoagulant	20 (87.0%)	85 (41.9%)	34 (39.1%)
Prophylactic-dose anticoagulant	0	68 (33.5%)	4 (4.6%)
Treatment-dose anticoagulant	0	0	36 (41.4%)
Died out of hospital	3 (13.0%)	50 (24.6%)	13 (14.9%)

© 2021 Vaughn VM et al. *JAMA Network Open.*

eTable 5. Cause of 60-Day Mortality, N=313

	**No Anticoagulation (N=23); N (%)**	**Prophylactic Only (N=203); N (%)**	**Treatment Dose Anticoagulants (N=87); N (%)**
COVID-19 (if COVID-19 selected)	13 (56.5%)	130 (64.0%)	69 (79.3%)
Cardiac causes (if fatal cardiac arrythmia, heart failure, myocardial infarction)	4 (17.4%)	24 (11.8%)	13 (14.9%)
Venous Thromboembolism	0 (0%)	1 (0.5%)	3 (3.5%)
Stroke	0 (0%)	2 (1.0%)	1 (1.2%)
Other infection (include other infection and c. diff)	1 (4.4%)	6 (3.0%)	3 (3.5%)
Pneumonia	11 (47.8%)	71 (35.0%)	42 (48.3%)
Combine refractory acidosis, refractory hypoxemia, refractory shock, and sepsis	11 (47.8%)	79 (38.9%)	41 (47.1%)
None of the above	4 (17.4%)	41 (20.2%)	7 (8.1%)

*may add up to >100% as patients could have multiple causes of death

© 2021 Vaughn VM et al. *JAMA Network Open.*

eTable 6. Sensitivity Analysis when 21 patients who did not receive treatment dose anticoagulation until after a confirmed VTE were classified as prophylactic-dose anticoagulation only

	Original Analysis: Adjusted Hazard Ratios (95% Confidence Intervals)		Sensitivity Analysis: Adjusted Hazard Ratios (95% Confidence Intervals)	
	**Inpatient Mortality**	**60-Day Mortality**	**Inpatient Mortality**	**60-Day Mortality**
Ever Treatment vs. Only Prophylactic-Dose Anticoagulants	1.036 (0.762, 1.410)	1.306 (0.987, 1.729)	1.204 (0.882,1.643)	1.417 (1.066, 1.883)
Ever Treatment vs. No Anticoagulant	0.377 (0.247, 0.575)	0.922 (0.631, 1.348)	0.413 (0.270, 0.630)	0.970 (0.662, 1.421)
Prophylactic Only vs. No Anticoagulant	0.364 (0.254, 0.518)	0.706 (0.514, 0.969)	0.343 (0.241,0.488)	0.685 (0.499, 0.938)

© 2021 Vaughn VM et al. *JAMA Network Open.*

eTable 7. VTE Location in Patients who Had a 60-day VTE Event

	No anticoagulation (N=0)	Prophylactic Only (N=16); N (%)	Treatment Dose Anticoagulation (N=32); N (%)
Any DVT	0	3 (18.8%)	20 (62.5%)
Any Proximal DVT	0	1 (6.3%)	11 (34.4%)
Only Distal DVT	0	1 (6.3%)	4 (12.5%)
UE DVT	0	0	1 (3.1%)
Unknown	0	1 (6.3%)	4 (12.5%)
PE	0	14 (87.5%)	30 (93.8%)

*may add up to >100% as patients could have multiple VTEs

© 2021 Vaughn VM et al. *JAMA Network Open.*

eTable 8. VTE Timing (out of all patients)

*numbers may not add up as patients may have multiple VTEs at multiple timepoints

	No anticoagulation (N=162)	Prophylactic Only (N=970); N (%)	Treatment Dose Anticoagulation (N=219); N (%)
PE within 60 days	0	11 (1.1%)	0 (0%)
DVT within 60 days	0	3 (0.3%)	1 (0.5%)
PE during hospitalization	0	3 (0.3%)	20 (9.1%)
DVT during hospitalization	0	0 (0%)	13 (5.9%)
PE or DVT ever during hospitalization or within 60 days	0	16 (1.7%)	32 (14.6%)

© 2021 Vaughn VM et al. *JAMA Network Open.*
